# Steroidal Saponins from *Vernonia amygdalina* Del. and Their Biological Activity

**DOI:** 10.3390/molecules23030579

**Published:** 2018-03-05

**Authors:** Jing Wang, Hua Song, Xiaoxue Wu, Shuyi Zhang, Xuemin Gao, Funan Li, Xuan Zhu, Qing Chen

**Affiliations:** Department of Pharmacy, School of Pharmaceutical Science, Xiamen University, Xiamen 361102, China; diaoli187@163.com (J.W.); songhua@xmu.edu.cn (H.S.); xiaoxue_wu1@126.com (X.W.); shuyi_zhang95@163.com (S.Z.); holygxm@xmu.edu.cn (X.G.); fnlee5@xmu.edu.cn (F.L.)

**Keywords:** *Vernonia amygdalina* Del., vernoniamyoside, chemical constituents, cytotoxic activity

## Abstract

In the present study, four new steroidal saponins, namely vernoniamyoside A–D (**1**–**4**), together with the two known steroidal saponins vernoamyoside D (**5**) and vernonioside B_2_ (**6**) were isolated from the ethanol extract of leaves of the African medicinal plant *Vernonia amygdalina* Del. (Asteraceae). Their structures were demonstrated by spectral analyses along with 1D and 2D nuclear magnetic resonance (NMR) techniques and mass spectrometry (MS). The cytotoxicity of the compounds was also tested by the 3-(4,5-dimethylthiazol-2-yl)-2,5-diphenyltetrazolium bromide (MTT) method on the cell lines Hela, MCF-7, BT-549 and MDA-MB-231. Vernoniamyoside A, vernoniamyoside B, and vernonioside B_2_ showed cytotoxicity towards BT-549 cell lines. Vernoniamyoside C, vernoniamyoside D and vernoamyoside D showed different levels of cytotoxic activities.

## 1. Introduction

*Vernonia amygdalina* Del. from the family Asteraceae is distributed throughout tropical Africa, especially in West Africa. It has received considerable scientific interest due to the observation that adult chimpanzees with malaria returned to normal activity after chewing the extract of the bitter juice of this species [1]. Over the years, several studies on the chemical components of this species, including flavonoids [2], sesquiterpene lactones [3], steroidal saponins [4,5,6], and fatty acids [7], have been performed. Previous studies have indicated different bioactivities of this species, including anti-inflammation [8], anti-malaria [9], anti-obesity [10,11], antioxidant [12,13], anti-tumor [14], and other activities [15]. Water and chloroform extracts of *V. amygdalina* (VA) interfere with the DNA synthesis of MCF-7 cells in breast cancer, affecting the activity of ERKs in vitro and inhibiting human breast cancer cells [16,17]. Via the MTT method, it has been verified that MCF-7 cells are inhibited in vitro and the DNA synthesis of BT-549 cells in breast cancer is disturbed by the addition of VA extract [18,19]. Moreover, three active fractions extracted from ethanol extracts of *V. amygdalina* have an inhibiting effect on tumor cells, the essential component being steroidal saponins [14]. So far, a certain amount of steroidal saponins has been obtained from *V. amygdalina;* however, studies on the anti-tumor activity are rare. It is therefore especially important to investigate the separation and biological activity of steroidal saponins in *V. amygdalina*. 

In the present study, four new steroidal saponins, vernoniamyoside A–D (**1**–**4**) along with two known steroidal saponins vernoamyoside D (**5**) and vernonioside B_2_ (**6**) were obtained. It is expected that these steroidal saponins also demonstrate anti-tumor activity, especially anti-breast cancer activity. Therefore, all compounds were evaluated for their cytotoxicity toward human Hela, MCF-7, BT-549, and MDA-MB-231 cell lines by means of the MTT method. The isolation, structure identification, and biological activities of the aforementioned compounds are described.

## 2. Results and Discussion

### 2.1. Isolation, Characterization, and Structure Elucidation of Compounds

Compound **1** was obtained as a white powder, and its molecular formula C_35_H_50_O_11_, determined by high resolution electronspray ionization-mass spectrum (HR-ESI-MS) at *m*/*z* 669.3240 [M + Na]^+^ (calcd for C_35_H_50_NaO_11_, 669.3206), has 11 degrees of unsaturation. In the ^1^H NMR spectrum of compound **1**, two olefinic proton signals at δ_H_ 5.40 (1H, br s, H-7) and 5.62 (1H, d, H-11), two angular methyl singlets at δ_H_ 0.52 (3H, s, CH_3_-18) and 0.87 (3H, s, CH_3_-19), two methyl doublets at δ_H_ 0.83 (3H, d, CH_3_-26), 0.91 (3H, d, CH_3_-27), and one methyl singlet 2.15 (3H, s, CH_3_-29) were observed. The ^13^C-NMR and distortionless enhancement by polarization transfer (DEPT) spectra indicated that compound **1** contained 35 carbon signals, including five methyls (at δ_C_ 13.8 (C-18), 19.4 (C-19), 17.1 (C-26), 17.0 (C-27) and 29.2 (C-29)), eight methines (including cyclic olefinic carbon signals at δ_C_ 121.9 (C-7), 133.1 (C-8), 143.9 (C-9), and 117.6 (C-11)), eight quaternary carbons (including two carbonyl carbon signals at δ_C_ 213.0 (C-28) and 214.2 (C-16) and a lactone carbon signal at δ_C_ 177.3 (C-21)), and 14 methylenes. Particularly, an obvious carbonyl signal at δ_C_ 214.2, corresponding to δ_H_ 2.63 (1H, H-17), 2.98 (H-20), 2.02 and 2.34 (H-15), in ^1^H-detected heteronuclear multiple-bond correlation (HMBC) was observed, suggesting a carbonyl group is connected to C-16. The ^1^H and ^13^C NMR analysis (see Table 1) supported the hypothesis that compound **1** might be Δ^7, 9 (11)^ stigmastane-type steroid derivative, with the same skeleton as vernonioside A_3_ [5]. Moreover, two carbon signals at δ_C_ 83.0 (C-24), 33.5 (C-25) and obvious HMBC correlations of δ_H_ 0.83 (3H, d, CH_3_-26) with δ_C_ 83.0, 33.5, 17.0 and δ_C_ 0.91 (3H, d, CH_3_-27) with δ_C_ 83.0, 33.5, 17.1 were observed. According to the NMR data and analysis, we suggest that compound **1** contains a-HOCCH (CH_3_)_2_ group. A carbonyl signal at δ_C_ 213.0 (C-28) also showed HMBC correlations with a methyl signal at δ_H_ 2.15 (3H, s, CH_3_-29). In the HMBC spectrum, δ_H_ 2.63 (H-17) has correlations of δ_C_ 26.0 (C-22), 36.8 (C-20), δ_H_ 4.63 (H-23) has correlations of δ_C_ 26.0 (C-22), 36.8 (C-20), 177.3 (C-21).

The typical carbon signal δ_C_ 100.9 has a ^1^H-detected heteronuclear single quantum correlation (HSQC) correlation of δ_H_ 4.24 (1H, d, H-1′) and in the HMBC spectrum, obvious correlations from δ_H_ 4.24 to δ_C_ 76.3 and δ_H_ 3.57 to δ_C_ 100.9 suggest a glucose moiety in compound **1**. The signal δ_C_ 100.9 is caused by anomeric carbon and the glucose moiety connected to C-3. The other carbon signals δ_C_ 61.1 and a series of signals between δ_C_ 70.0 and 80.0, along with proton signals between δ_H_ 2.90 and 3.90, are a better validation of the previous assumption. Through acid hydrolysis and a comparison of the retention time to standard d-glucose, the glucose moiety was confirmed to be d-glucose. Compared to the known compounds, compound **1** has the same side chain as vernoamyoside D [6].

**Figure 1:**
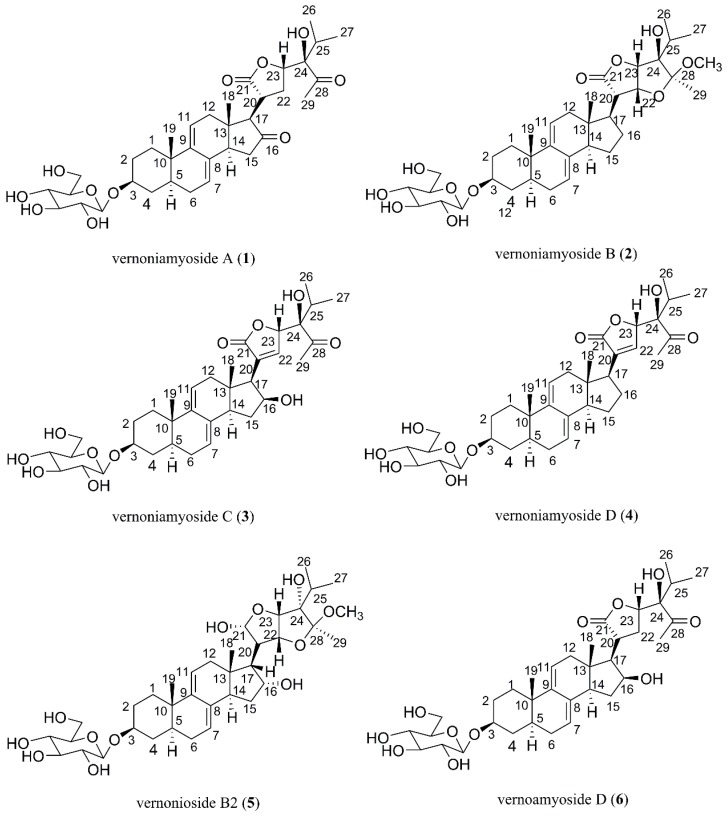
Structures of compounds **1**–**6**.

**Figure 2:**
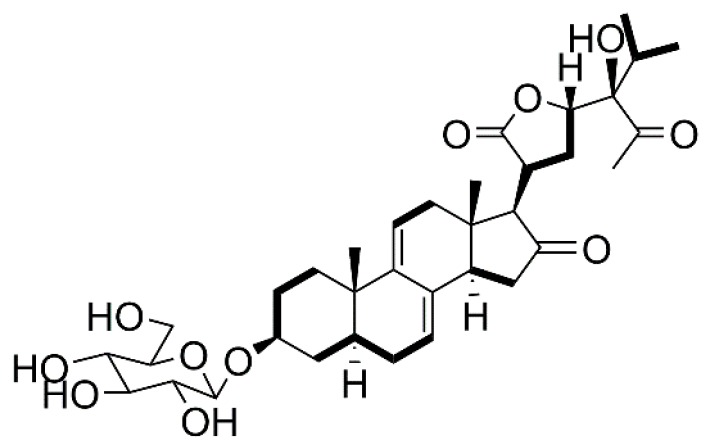
Major ^1^H-^1^H correlation spectroscopy (^1^H-^1^H COSY) correlations of compound **1** (bold lines).

**Figure 3:**
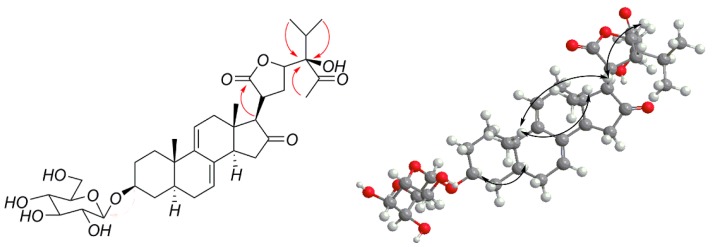
Major HMBC and NOESY correlations of compound **1**.

First, the planar structure of compound **1** was determined. The nuclear overhauser effect spectroscopy (NOESY) correlation between H-3 (δ_H_ 3.57) and H-5 (δ_H_ 1.35), H-5 (δ_H_ 1.35) and H-14 (δ_H_ 2.65), Me-18 (δ_H_ 0.52) and Me-19 (δ_H_ 0.87), as well as Me-18 (δ_H_ 0.52) and H-20 (δ_H_ 2.98) indicated that compound **1** contains trans rings of nuclear parent and H-3, H-5 and H-14 were α-configurated, while Me-18, Me-19, and H-20 were β-configurated. A NOESY correlation from H-17 (δ_H_ 2.63) to H-14 (δ_H_ 2.65) was observed, which determined that H-17 has an α-orientation. Peaks from H-23 (δ_H_ 4.63) to H-20 (δ_H_ 2.98) indicated that the proton H-23 was β-configurated. The configuration of C-24 could not be determined by NOESY; thus, we compared the carbon signals between compound **1** and vernoamyoside D [6] with the same fragment. Thus, compound **1** was determined and named vernoniamyoside A (see Figure 1, Figure 2 and Figure 3).

Compound **2** was obtained as a white powder, and its molecular formula C_36_H_54_O_11_, determined by HR-ESI-MS at *m*/*z* 685.3554 [M + Na]^+^, (calcd for C_36_H_54_NaO_11_, 685.3553), has 10 degrees of unsaturation. The ^1^H NMR spectrum of compound **2** showed the following specific signals: two methyl doublets at δ_H_ 0.87 (3H, d, CH_3_-26), 1.03 (3H, d, CH_3_-27), and two methyl singlets at 1.26 (3H, s, CH_3_-29) and 3.15 (3H, s, OCH_3_). The ^13^C NMR spectra indicated 36 carbon signals and showed the following typical five methyl signals: (at δ_C_ 11.8 (C-18), 19.4 (C-19), 16.5 (C-26), 17.6 (C-27), and 15.0 (C-29)). In the HMBC spectrum, we observed correlations between δ_H_ 1.39 and 1.76 with δ_C_ 22.8 (C-16), δ_H_ 1.39 with δ_C_ 28.0 (C-15), 50.9 (C-17), 135.8 (C-8), and δ_H_ 1.76 with δ_C_ 41.8 (C-13), 50.9 (C-17), indicating that the signal δ_C_ 22.8 is connected to C-16. We therefore infer that compound **2** is Δ^7, 9 (11)^, a stigmastane-type steroid derivative with the same skeleton as vernonioside B1 [1] by comparison with ^1^H-NMR and ^13^C-NMR (see Table 1).

**Figure 4:**
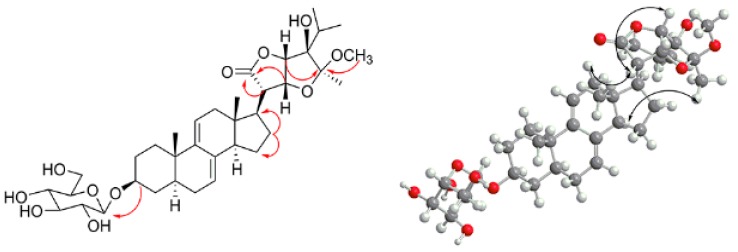
Major HMBC and NOESY correlations of compound **2**.

A large number of ^13^C-NMR signals were between δ_C_ 70.0 and 90.0, indicating that there is a highly oxidized side chain. In the HMBC spectrum, the correlations of H-25 (δ_H_ 1.91), H-26 (δ_H_ 0.87), and H-27 (δ_H_ 1.03) to the carbon signals at δ_C_ 82.6 (C-24) and H-22 (δ_H_ 4.58), H-23 (δ_H_ 4.62), H-29 (δ_H_ 1.26) to δ_C_ 106.9 (C-28), and H-22 (δ_H_ 4.58), H-23 (δ_H_ 4.62), H-20 (δ_H_ 2.76) to δ_C_ 175.4 (C-21) indicate that the side chain is similar compared to that of vernoamyoside C [6]. The difference between compound **2** and vernoamyoside C is C-21, the absence of signals at δ_H_ 5.45 and δ_C_ 100.0, and the presence of signals at δ_C_ 175.4, indicating that the side chain is highly oxidized. This helped us to determine the planar structure of compound **2**. The correlation between H-3 (δ_H_ 3.56) and H-5 (δ_H_ 1.29), H-5 (δ_H_ 1.29) and H-14 (δ_H_ 1.94), Me-18 (δ_H_ 0.54) and Me-19 (δ_H_ 0.85), and Me-18 (δ_H_ 0.54) and H-20 (δ_H_ 2.76) indicated that compound **2** has the trans rings of the nuclear parent and H-3, H-5 and H-14 adopted an α-configuration, while Me-18, Me-19 and H-20 were β-configurated. This is similar to compound **1**. The NOESY spectrum from H-17 (δ_H_ 2.19) to H-14 (δ_H_ 1.94) was observed, which determined that the H-17 has α-orientation. Peaks from H-23 (δ_H_ 4.62) to H-20 (δ_H_ 2.76) indicated that proton H-23 was β-configurated. The significant signals from H-20 (δ_H_ 2.76) to H-18 (δ_H_ 0.54), H-22 (δ_H_ 4.58) and H-23 (δ_H_ 4.62) indicated that H-22 (δ_H_ 4.58) and H-23 (δ_H_ 4.62) were β-configurated. The correlation between H-29 (δ_H_ 1.26) and H-14 (δ_H_ 1.94) suggested that CH_3_-29 has an *α*-configuration [6]. Thus, compound **2** was determined and named vernoniamyoside B (see Figure 1 and Figure 4).

**Figure 5:**
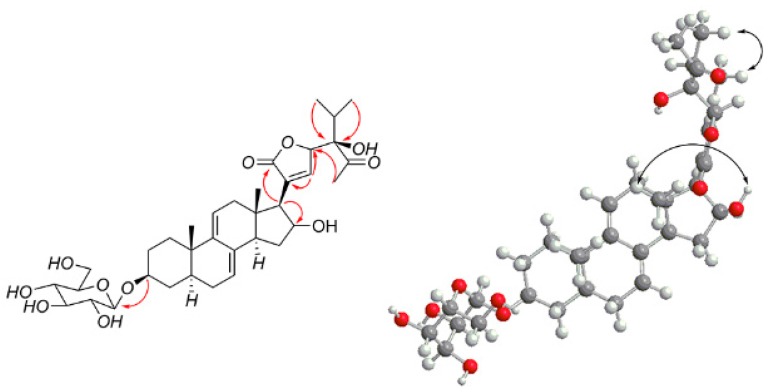
Major HMBC and NOESY correlations of compound **3**.

Compound **3** was obtained as a white powder, and its molecular formula C_35_H_50_O_11_, determined by HR-ESI-MS at *m*/*z* 669.3250 [M + Na]^+^ (calcd for C_35_H_50_NaO_11_, 669.3206), has 11 degrees of unsaturation. The proton signal is at δ_H_ 4.40 (H-16), which has correlation with δ_C_ 48.7 (C-14) in the HMBC spectrum. The correlations between H-17 (δ_H_ 2.41) and the carbon signal at δ_C_ 74.3 in the HMBC spectrum confirm that the δ_C_ 74.3 is connected to C-16. The ^1^H NMR and ^13^C NMR signals (see Table 1) of compound **3** indicated that it is a Δ^7, 9 (11)^ stigmastane-type steroid derivative with the same skeleton as vernoamyoside D [6]. According to the HMBC, the signal at δ_H_ 7.49 has a correlation with δ_C_ 56.6 (C-17), 173.3 (C-21), 82.8 (C-23), while δ_H_ 2.41 (H-17) and 5.24 (H-23) have correlations with δ_C_ 133.9. Therefore, there is a double bond between C-20 (δ_C_ 133.9) and C-22 (δ_C_ 146.8). Thus, the planar structure of compound **3** could be determined. Compared to the ^1^H NMR and ^13^C NMR signals, the side chain is the same as in vernonioside A4 [20]. The significant nuclear overhauser effect (NOE) correlations between H-16 (δ_H_ 4.40) and Me-18 (δ_H_ 0.30) suggested that OH-16 has an β-configuration. Therefore, compound **3** was determined and named vernoniamyoside C (see Figure 1 and Figure 5).

**Figure 6:**
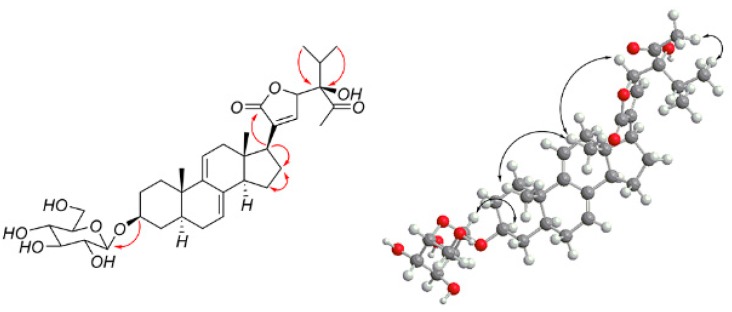
Major HMBC and NOESY correlations of compound **4**.

Compound **4** was obtained as a white powder, and its molecular formula C_35_H_50_O_10_, determined by HR-ESI-MS at *m*/*z* 653.3303 [M + Na]^+^ (calcd for C_35_H_50_NaO_10_, 653.3257), has 11 degrees of unsaturation. The ^13^C NMR spectra indicated 35 carbon signals. Compared with the ^1^H NMR and ^13^C NMR signals (see Table 1) of compound **2**, compound **4** is also a Δ^7, 9 (11)^ stigmastane-type steroid derivative with a glycoside. As for the side chain, δ_H_ 5.22 (H-23) has correlations with δ_C_ 135.7 in the HMBC spectrum. Compound **4** had similar ^1^H, ^13^C NMR, and NOESY data when compared with compound **3**. Thus, compound **4** was determined and named vernoniamyoside D (see Figure 1 and Figure 6).

According to the literature, the two known compounds are identified as vernoamyoside D (**5**) [6] and vernonioside B_2_(**6**) [20] (see Figure 1).

### 2.2. Results of the Cytotoxicity Test

According to a previous study, MCF-7 cells are inhibited in vitro, and the DNA synthesis of BT-549 cells in breast cancer is disturbed by the application of *V. amygdalina* extract [18,19]. A series of steroidal saponins exhibited anti-tumor activity, especially anti-breast tumor activity [14]. In this study, the cytotoxicity of compounds **1**–**6** was tested on BT-549, MDA-MB-231, MCF-7, and Hela cell lines. The anti-tumor activity of Δ^7, 9 (11)^ stigmastane-type steroidal saponins was introduced for the first time. As seen in Table 2, the inhibition against the BT-549 cell line of compound **1** could reach up to 63.61%, while compound **2** and **6** also showed cytotoxicity towards the BT-549 cell line (inhibition = 62.17 and 51.14%). This leads us to infer that compounds **1**, **2**, and **6** are highly toxic towards BT-549 cell lines, while they showed a general cytotoxicity to cell lines MDA-MB-231, MCF-7, and Hela. Based on this, these compounds might play a certain role in the treatment of breast cancer. The cytotoxicity activities of compound **3**, **4**, and **5** showed different levels against the tested cell lines. Moreover, further studies are necessary to confirm whether these compounds are also toxic towards other tumor cell lines. Both compounds have the same sugar chains; therefore, the different activities might be due to the side chain and the nuclear parent. Furthermore, it should be highlighted that compounds **1**-**6** was had a different selectivity for tumor cell lines. 

## 3. Materials and Methods

### 3.1. General Experimental Procedures

Optical rotations were measured with an Automatic polarimeter (Hackettstown, NJ, USA). UV spectra were recorded by a Shimadzu UV-2600 PC spectrophotometer (Suzhou, Jiangsu, China). IR (KBr-disks) spectra were measured using a Bruker Alpha (Karlsruhe, Germany). The HR-ESI-MS spectra were recorded by a Thermo Scientific Q Exactive Plus Orbitrap LC-MS/MS system (Waltham, MA, USA); NMR spectra were recorded by a Bruker AVANCE III 600 MHz spectrometer (Zurich, Switzerland) in CD_3_COCD_3_, with TMS as internal standard. The preparative high-performance liquid chromatography (HPLC) system consisted of an LC-6AD intelligent prep. pump (Kyoto, Japan), an SPD-20A intelligent UV/VIS detector (Kyoto, Japan), and a YMC-Park ODS-A column (5 μm, 250 × 10 mm I.D., YMC Co. Ltd., Ishikawa, Japan). Silica gel GF_254_ for thin-layer chromatography (TLC) and silica gel (200–300 mesh) for column chromatography (CC) were obtained from Qingdao Marine Chemical Factory (Qingdao, Shandong, China). Sephadex LH-20 (Merck, Darmstadt, Germany) was suited for size-exclusion chromatography. The cell lines BT-549 Hela, MCF-7, and MDA-MB-231 were obtained from China Center for Type Culture Collection (Wuhan, Hubei, China); MTT were obtained from Sigma Company. All solvents were purchased from Sinopharm Chemical Reagents (Shanghai, China). Methyl alcohol used for HPLC analysis was of chromatographic grade (Sigma, St. Louis, MO, USA). All aqueous solutions were prepared with double-distilled water.

### 3.2. Plant Material

Dried leaves of *V. amygdalina* were collected in Xiamen, Fujian Province, China, and identified by Qing Chen, Assistant Professor from the School of Pharmaceutical Sciences, Xiamen University. 

### 3.3. Extraction and Isolation 

The dried leaves (7.5 kg) were extracted with 95% ethanol and concentrated under vacuum to obtain crude extract. Subsequently, the crude extract was suspended in H_2_O and partitioned in petroleum ether, dichloromethane (CH_2_Cl_2_), ethyl acetate, and *n*-butanol in sequence to obtain the petroleum ether fraction (170 g), the dichloromethane fraction (224 g), the ethyl acetate fraction (80 g), and the *n*-butanol fraction (60 g), respectively. The dichloromethane fraction (224 g), a dark green syrup, was subjected to macroporous resin column chromatography (CC) (4 kg, D101) and eluted with gradient methyl alcohol (MeOH)-H_2_O (8:2) and MeOH. After removing the solvents under vacuum, the MeOH-H_2_O extract (110 g) was subjected to silica gel column chromatography (CC) (1 kg, 200–300 mesh) and eluted with gradient CH_2_Cl_2_-MeOH (50:1 to 1:1 *v*/*v*) and MeOH to obtain fractions A (9.8 g), B (14.2 g), C (21.7 g), D (22.2 g), E (15.7 g), F (14.7 g), and G (8.5 g) after deducting the solvents. Fraction C was re-chromatographed on a silica gel CC eluted with CH_2_Cl_2_-MeOH, using a gradient (15:1 to 5:1 *v*/*v*) to obtain five fractions (Ca-Ce). Fraction Cb was further purified by Sephadex LH-20 eluted with MeOH to obtain 32 fractions, which were pooled together using TLC to obtain the three sub-fractions Cba-Cbc. The fraction Cbb (922 mg) was separated by preparative HPLC, using a YMC-Park ODS-A column (5 μm, 250 × 10 mm I.D.) with a flow rate of 5 mL/min and a mobile phase of MeOH-H_2_O (75:25) to obtain the five sub-fractions Cbba-Cbbe. Sub-fraction Cbba (100 mg) was purified by preparative HPLC, using a YMC-Park ODS-A column (5 μm, 250 × 10 mm I.D.) with a flow rate of 5 mL/min and a mobile phase of MeOH-H_2_O (58:42) to yield compound **1** (49 mg). Sub-fraction Cbbd (18 mg) was purified by preparative HPLC, using a YMC-Park ODS-A column (5 μm, 250 × 10 mm I.D.) with a flow rate of 5 mL/min and a mobile phase of MeOH-H_2_O (70:30) to yield compound **4** (5.1 mg). Sub-fraction Cbbe (40 mg) was purified by preparative HPLC, using a YMC-Park ODS-A column (5 μm, 250 × 10 mm I.D.) with a flow rate of 5 mL/min and a mobile phase of MeOH-H_2_O (75:25) to yield compound **2** (21 mg). Fraction D was re-chromatographed on silica gel CC eluted with CH_2_Cl_2_-MeOH, using a gradient (12:1 to 1:1 *v*/*v*) which was pooled together using TLC to obtain six fractions (Da-Df). Fraction Dd (828 mg) was purified by preparative HPLC, using a YMC-Park ODS-A column (5 μm, 250 × 10 mm I.D.) with a flow rate of 5 mL/min and a mobile phase of MeOH-H_2_O (57:43) to yield compounds **3** (22 mg) and **5** (16 mg). Fraction Db was further purified by preparative HPLC, using a YMC-Park ODS-A column (5 μm, 250 × 10 mm I.D.) with a flow rate of 5 mL/min and a mobile phase of MeOH-H_2_O (71:29) to obtain two sub-fractions. Sub-fraction Dbb (40 mg) was further purified by preparative HPLC, using a YMC-Park ODS-A column (5 μm, 250 × 10 mm I.D.) with a flow rate of 5 mL/min and a mobile phase of MeOH-H_2_O (73:27) to obtain compound **6** (27 mg).

#### 3.3.1. Vernoniamyoside A (**1**)

White amorphous powder; HR-ESI-MS *m*/*z* 669.3240 [M + Na]^+^ (calcd for C_35_H_50_NaO_11_, 669.3206); [α${]}_{D}^{25}$: −0.32 (c 0.05, CH_3_OH); UV (DMSO): λmax: 252.0, 208.8 nm; IR (KBr) *v*_max_: 3392, 2933, 2361, 1770, 1704, 1354, 1025 cm^−1^; ^1^H and ^13^C NMR data: see Table 1.

#### 3.3.2. Vernoniamyoside B (**2**)

White amorphous powder; HR-ESI-MS *m*/*z* 685.3554 [M + Na]^+^ (calcd for C_36_H_54_NaO_11_, 685.3553); [α${]}_{D}^{25}$: +0.40 (c 0.10, CH_3_OH); UV (DMSO): λmax: 252.0, 207.2 nm; IR (KBr) *v*_max_: 3395, 2937, 2360, 1775, 1382, 1025 cm^−1^; ^1^H and ^13^C NMR data: see Table 1.

#### 3.3.3. Vernoniamyoside C (**3**)

White amorphous powder; HR-ESI-MS *m*/*z* 669.3250 [M + Na]^+^ (calcd for C_35_H_50_NaO_11_, 669.3206); [α${]}_{D}^{25}$: *−*0.20 (c 0.05, CH_3_OH); UV (DMSO): λmax: 252.0, 208.8 nm; IR (KBr) *v*_max_: 3735, 3421, 2933, 2361, 1750, 1716, 1507, 1024 cm^−1^; ^1^H and ^13^C NMR data: see Table 1.

#### 3.3.4. Vernoniamyoside D (**4**)

White amorphous powder; HR-ESI-MS *m*/*z* 653.3303 [M + Na]^+^ (calcd for C_35_H_50_NaO_10_, 653.3257); [α${]}_{D}^{25}$: −0.24 (c 0.05, CH_3_OH); UV (DMSO): λmax: 252.0, 208.8 nm; IR (KBr) *v*_max_: 3735, 3421, 2933, 2361, 1750, 1716, 1558, 1507, 1024 cm^−1^; ^1^H and ^13^C NMR data: see Table 1.

#### 3.3.5. Vernoamyoside D (**5**)

White amorphous powder; ^1^H and ^13^C NMR data comparable to published data [6]. ^13^H NMR (600Hz, CD_3_COCD_3_): δ_H_ 5.33 (1H, br s, H-7) and 5.51 (1H, d, H-11),0.44 (3H, s, CH_3_-18), 0.83 (3H, s, CH_3_-19), 0.84 (3H, d, CH_3_-26), 0.91 (3H, d, CH_3_-27) and 2.16 (3H, s, CH_3_-29). ^13^C NMR (150Hz, CD_3_COCD_3_): δ_C_ 33.7 (C-1),29.3 (C-2), 76.3 (C-3), 34.2 (C-4), 38.7 (C-5), 29.8 (C-6), 121.9 (C-7), 135.1(C-8), 143.8 (C-9), 35.6 (C-10), 118.8 (C-11), 40.7 (C-12), 42.4 (C-13), 48.1 (C-14), 34.0 (C-15), 73.5 (C-16), 59.1 (C-17), 12.8 (C-18), 19.4 (C-19), 39.4 (C-20), 177.8 (C-21), 27.4 (C-22), 80.4 (C-23), 83.8 (C-24), 33.4 (C-25), 17.2 (C-26), 17.0 (C-27), 213.5 (C-28), 29.4 (C-29), 100.9 (C-1′), 73.5 (C-2′), 76.7 (C-3′), 70.1 (C-4′), 76.7 (C-5′), 61.2 (C-6′).

#### 3.3.6. Vernonioside B_2_ (**6**)

White amorphous powder; ^1^H and ^13^C NMR data: comparable to published data [20]. ^13^H NMR (600Hz, CD_3_COCD_3_): δ_H_ 5.36 (1H, br s, H-7) and 5.47 (1H, d, H-11),0.48 (3H, s, CH_3_-18), 0.83 (3H, s, CH_3_-19), 0.85 (3H, d, CH_3_-26), 0.88 (3H, d, CH_3_-27), 1.33 (3H, s, CH_3_-29) and 3.12 (3H, s, OCH_3_). ^13^C NMR (150Hz, CD_3_COCD_3_): 35.2 (C-1), 29.7 (C-2), 77.1 (C-3), 34.9 (C-4), 38.9 (C-5), 29.9 (C-6), 121.8 (C-7), 135.7 (C-8), 143.7(C-9), 36.0 (C-10), 118.6 (C-11), 41.1 (C-12), 43.1 (C-13), 48.5 (C-14), 34.9 (C-15), 76.7 (C-16), 54.9 (C-17), 14.3 (C-18), 19.7 (C-19), 47.9 (C-20), 98.4 (C-21), 80.2 (C-22), 90.2 (C-23), 81.3 (C-24), 31.7 (C-25), 17.5 (C-26), 18.4 (C-27), 112.4 (C-28), 17.5 (C-29), 101.4 (C-1′), 75.5 (C-2′), 80.2 (C-3′), 72.9 (C-4′), 77.2 (C-5′), 61.6 (C-6′), 48.2 (OCH_3_).

### 3.4. Acid Hydrolysis of Compounds

Compounds **1**–**4** (1 mg each) were dissolved in 2 M HCl (5.0 mL) and stirred at 90°C for 4 h. The reaction solution was neutralized with NH_4_OH and partitioned between EtOAc and H_2_O. The residue was obtained from the water layer under vacuum distillation and dissolved in pyridine (1 mL), which was added to 0.1 M l-cysteine methyl ester hydrochloride in pyridine (1 mL). After being heated to 60 °C for 1 h, the mixture was added to phenyl isothiocynate in pyridine (1 mL) and stirred at 60 °C for an additional 1 h. After removal of the solvent, the residue was dissolved in MeOH and analyzed by HPLC, with the mobile phase CH_3_CN-H_2_O (30:80, *v*/*v*) containing 0.1% formic acid; the flow rate was 1 mL/min. The standard d-glucose derivative was prepared in the same way. We then compared the retention times of sugar derivatives obtained from compounds with standard d-glucose derivatives (d-glucose: 19.5 min).

### 3.5. Cytotoxicity Assay

The cytotoxicity of compounds **1**–**6** was tested on cell lines MCF-7, BT-549, MDA-MB-231, and Hela via the MTT method. Cells were seeded into 96-well microplates at 100 μL per well (MCF-7, MDA-MB-231, and A549 were cultured in Dulbecco’s modified eagle medium (DMEM) supplemented with 10% fetal calf serum and 1% penicillin-streptomycin solution, while BT-549 was cultured in Roswell Park Memorial Institute (RPMI) (according to previous experiments, the DMSO concentration we used showed no interference with the experimental results). After 48 h, we added MTT (0.5 mg/mL) dissolved in 100 μL of fetal calf serum per well. After 4 h, we removed the MTT reagent and added 150 μL DMSO to each well. Doxorubicin was used as the positive control. The sample without test compounds was used as the negative control. Absorbance was measured at 490 nm in an automated microplate reader. All experiments were performed in triplicate. Inhibition was calculated via the following equation:

$$\mathrm{Inhibition}=\left[\frac{A_{test\;sample}-A_{blank}}{A_{negative\;control}-A_{blank}}\right]\times 100\%$$

*A_test sample_* is the test sample absorbance, *A_blank_* is the blank absorbance, *A_negative control_* is the negative control absorbance.

## 4. Conclusions

We obtained four new steroidal saponins, namely vernoniamyoside A–D (**1**–**4**), together with the two known steroidal saponins vernoamyoside D (**5**) and vernonioside B_2_ (**6**) from *V. amygdalina*. Of these, **1**, **2**, and **6** showed an excellent cytotoxicity on BT-549 cell lines in the cytotoxicity activity assay (Table 2), as expected. It is worth noting that **1** and **2** were selective for different tumor cell lines. Our results indicate that substances **1** and **2** were toxic to BT-549 cell lines. Further studies should consider the screening of more types of tumor cells. 

The novelty of the saponins **1**–**4** is represented by the highly oxidized groups in the side chain, which may be associated with their cytotoxic expression. Saponin **1** has a strong cytotoxicity to BT-549 cell lines, including two ketones and one ester group. It is assumed that the cytotoxicity of saponin **1** may be derived from the C=O group. This group, present in the highly oxidized side chains of saponins **2**–**4,** may provide a basis for the cytotoxicity for BT-549. However, the different inhibitory effects may be due to the relationships between the different structures and spatial configurations. The specific mechanisms and verifications offer a new direction for further experiments.

The separated steroidal saponins from the dichloromethane extraction of *V. amygdalina*, are mainly hypoglycemic steroidal saponins. However, it is essential to separate polysaccharide steroidal saponins, which always exist in ethyl acetate and *n*-butanol extraction, from *V. amygdalina*. In addition to the steroidal saponins, *V. amygdalina* contains a series of active substances such as sesquiterpene lactones and flavonoids. Therefore, the separation and identification of these chemical components should be one of the most promising trends in the study of *V. amygdalina*.

Our results provide a scientific basis for the use of *V. amygdalina* in anti-tumor research, with a potential value in the treatment of cancer.

## Figures and Tables

**Table 1:** ^1^H (600 MHz) and ^13^C (150 MHz) NMR data of **1**–**4** in CD_3_COCD_3_.

No.	1 ^a^		2 ^a^		3 ^a^		4 ^a^	
	δ_C_	δ_H_ (*J* in Hz)	δ_C_	δ_H_ (*J* in Hz)	δ_C_	δ_H_ (*J* in Hz)	δ_C_	δ_H_ (*J* in Hz)
1	34.1	1.27, 2.0, m	34.3	1.23, 1.96, m	34.3	1.24, 1.93, m	34.3	1.24, 1.94, m
2	29.2	1.48, 1.88, m	29.3	1.45, 1.87, m	29.2	1.44, 1.84,m	29.2	1.46, 1.87, m
3	76.3	3.57, m	76.3	3.56, m	76.2	3.56, m	76.2	3.56, m
4	33.7	1.22, d(11.6)	33.7	1.19, d(11.6)	33.7	1.18, d(11.7)	33.7	1.18, d(11.7)
		1.83, m		1.80, m		1.79, m		1.76, m
5	38.7	1.35 ^a^	38.7	1.29 ^a^	38.6	1.31 ^a^	38.6	1.31 ^a^
6	29.4	1.26, 1.88 ^a^	29.5	1.23, 1.87 ^a^	29.5	1.24, 1.84 ^a^	29.4	1.23, 1.87 ^a^
7	121.9	5.40, br s	120.1	5.37, br s	120.7	5.36, br s	120.7	5.39, br s
8	133.1		135.8		135.1		135.7	
9	143.9		143.3		143.6		143.7	
10	35.8		35.5		35.6		35.9	
11	117.6	5.62, d(5.8)	119.0	5.53, d(6.2)	117.9	5.47 ^a^	118.3	5.51 d(6.2)
12	39.0	2.27, m	40.8	2.01, d(7.4)	39.6	1.83, d(6.6)	39.4	1.94, d(6.6)
				2.84, m		2.17, m		2.16, m
13	40.3		41.8		43.6		43.4	
14	45.0	2.65 ^a^	45.1	1.94 ^a^	48.7	2.54 ^a^	46.0	2.52 ^a^
15	37.0	2.02, 2.34 ^a^	28.0	1.47, 2.09 ^a^	34.5	1.66, 1.92 ^a^	26.5	1.91 ^a^
16	214.2		22.8	1.39, 1.76 ^a^	74.3	4.40 ^a^	23.1	1.49, 1.85 ^a^
17	61.2	2.63, d(2.9)	50.9	2.19 ^a^	56.6	2.41, d(7.5)	50.8	2.27 ^a^
18	13.8	0.52, s, 3H	11.8	0.54, s, 3H	13.2	0.30, s, 3H	12.1	0.32, s, 3H
19	19.4	0.87, s, 3H	19.4	0.85, s, 3H	19.3	0.81, s, 3H	19.3	0.82, s, 3H
20	36.8	2.98, ddd, (12.2,8.7,3.2)	47.6	2.76, dd,	133.9		134.9	
				(10.8, 6.6)				
21	177.3		175.4		173.3		172.9	
22	26.0	1.88, 2.16 ^a^	79.1	4.58, t(6.0*2)	146.8	7.49, br s	146.9	7.44, br s
23	80.6	4.63, d(5.5)	78.7	4.62, d(5.1)^,^	82.8	5.24, m	82.7	5.22, m
24	83.0		82.6		83.3		83.3	
25	33.5	2.07, m	30.8	1.91, m	32.9	2.16, m	32.8	2.16, m
26	17.1	0.83, d(7.0)	16.5	0.87, d(6.8)	16.9	0.82, d(6.9)	16.9	0.81, *d*(6.8)
27	17.0	0.91, d(7.0)	17.6	1.03, d(6.8)	16.7	0.97, d(6.9)	16.6	0.96, *d*(6.8)
28	213.0		106.9		211.2		211.1	
29	29.2	2.15, s, 3H	15.0	1.26, s, 3H	28.1	2.09, s, 3H	28.1	2.09, s, 3H
Glu								
1′	100.9	4.24, d(8.1)	100.9	4.23, d(8.1)	100.9	4.22, d(8.0)	100.9	4.22, d(8.1)
2′	73.5	2.90 ^a^	73.5	2.90 ^a^	73.5	2.89 ^a^	73.5	2.89 ^a^
3′	76.7	3.10 ^a^	76.7	3.10 ^a^	76.8	3.10 ^a^	76.8	3.11 ^a^
4′	70.1	3.03 ^a^	70.1	3.02 ^a^	70.1	3.02 ^a^	70.1	3.02 ^a^
5′	76.8	3.10 ^a^	76.8	3.10 ^a^	76.8	3.10 ^a^	76.8	3.11 ^a^
6′	61.1	3.42, 3.65 ^a^	61.1	3.41, 3.65 ^a^	61.2	3.41, 3.65 ^a^	61.1	3.41, 3.65 ^a^
OCH_3_			49.5	3.15, s, 3H				

^a^ Resonance pattern unclear due to overlapping.

**Table 2:** Results of the cytotoxicity assay.

Sample	BT-549		MDA-MB-231		MCF-7		Hela	
	Average Absorbance	Inhibition (%)	Average Absorbance	Inhibition (%)	Average Absorbance	Inhibition (%)	Average Absorbance	Inhibition (%)
Compound **1**	0.2748	63.61	0.6102	28.97%	0.4217	46.54	0.5212	42.05
Compound **2**	0.2836	62.17	0.6196	27.78%	0.4893	37.07	0.6063	31.64
Compound **3**	0.4546	34.18	0.5803	32.74%	0.4728	39.38	0.6464	26.73
Compound **4**	0.3946	44.00	0.5899	31.53%	0.5301	31.36	0.5957	32.93
Compound **5**	0.4410	36.41	0.5734	33.61%	0.3990	49.72	0.6893	21.48
Compound **6**	0.3510	51.14	0.5961	30.75%	0.4750	39.08	0.5737	35.63
Positive control	0.1515	83.79	0.1789	83.39%	0.0734	95.32	0.0996	92.70
Negative control	0.6634		0.8399		0.7540		0.8648	
Blank	0.0525		0.0470		0.0400		0.0477	

## Supplementary Materials

The following ^1^H NMR, ^13^C NMR, 2D-NMR, and HR-ESI-MS spectra are available as supporting data. Supplementary materials are available online.
