# Supplementary figures and images for: Steroidal Saponins from Vernonia amygdalina Del. and Their Biological Activity
Source: Molecules. 2018 Mar 5;23(3):579. doi: 10.3390/molecules23030579 (PMC6017044; doi:10.3390/molecules23030579)

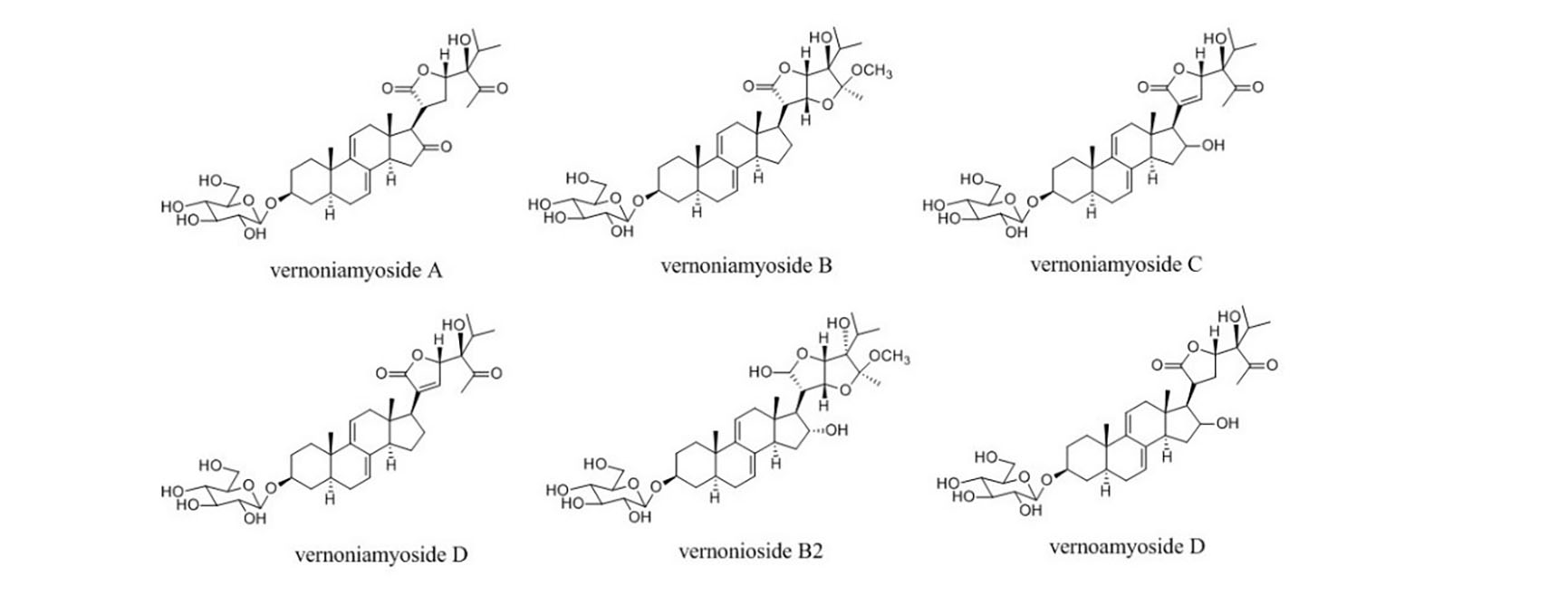

Supplement: Supplementary file 1 [file molecules-23-00579-s001.zip › molecules-269783-graphical.jpg]
