# Supplementary material for: Steroidal Saponins from Vernonia amygdalina Del. and Their Biological Activity
Source: Molecules. 2018 Mar 5;23(3):579. doi: 10.3390/molecules23030579 (PMC6017044; doi:10.3390/molecules23030579)
Supplement: Supplementary file 1 [file molecules-23-00579-s001.zip › molecules-269783-figures.zip!/tables and figures.docx]

**Tables**

Table 1 ^1^H (600 MHz) and ^13^C (150 MHz) NMR data of **1-4** in **CD_3_COCD_3_**

| No. | 1^a^ |  |  | 2^a^ |  |  | 3^a^ |  |  | 4^a^ |  |
| --- | --- | --- | --- | --- | --- | --- | --- | --- | --- | --- | --- |
|  | *δ*C | *δ*_H (_J in Hz) |  | *δ*C | *δ*_H (_J in Hz) |  | *δ*C | *δ*_H (_J in Hz) |  | *δ*C | *δ*_H (_J in Hz) |
| 1 | 34.1 | 1.27, 2.0, m |  | 34.3 | 1.23, 1.96, m |  | 34.3 | 1.24, 1.93, m |  | 34.3 | 1.24, 1.94, m |
| 2 | 29.2 | 1.48,1.88,m |  | 29.3 | 1.45,1.87, m |  | 29.2 | 1.44,1.84,m |  | 29.2 | 1.46,1.87, m |
| 3 | 76.3 | 3.57, m |  | 76.3 | 3.56, m |  | 76.2 | 3.56, m |  | 76.2 | 3.56, m |
| 4 | 33.7 | 1.22,*d*(11.6) |  | 33.7 | 1.19, *d*(11.6) |  | 33.7 | 1.18, *d*(11.7) |  | 33.7 | 1.18, *d*(11.7) |
|  |  | 1.83, m |  |  | 1.80, m |  |  | 1.79, m |  |  | 1.76, m |
| 5 | 38.7 | 1.35^a^ |  | 38.7 | 1.29^a^ |  | 38.6 | 1.31^a^ |  | 38.6 | 1.31^a^ |
| 6 | 29.4 | 1.26, 1.88^a^ |  | 29.5 | 1.23, 1.87^a^ |  | 29.5 | 1.24, 1.84^a^ |  | 29.4 | 1.23, 1.87^a^ |
| 7 | 121.9 | 5.40, *br s* |  | 120.1 | 5.37, *br s* |  | 120.7 | 5.36, *br s* |  | 120.7 | 5.39, *br s* |
| 8 | 133.1 |  |  | 135.8 |  |  | 135.1 |  |  | 135.7 |  |
| 9 | 143.9 |  |  | 143.3 |  |  | 143.6 |  |  | 143.7 |  |
| 10 | 35.8 |  |  | 35.5 |  |  | 35.6 |  |  | 35.9 |  |
| 11 | 117.6 | 5.62, *d* (5.8) |  | 119.0 | 5.53, *d* (6.2) |  | 117.9 | 5.47^a^ |  | 118.3 | 5.51 *d* (6.2) |
| 12 | 39.0 | 2.27,m |  | 40.8 | 2.01, *d*(7.4) |  | 39.6 | 1.83, *d*(6.6) |  | 39.4 | 1.94, *d*(6.6) |
|  |  |  |  |  | 2.84, m |  |  | 2.17, m |  |  | 2.16, m |
| 13 | 40.3 |  |  | 41.8 |  |  | 43.6 |  |  | 43.4 |  |
| 14 | 45.0 | 2.65^a^ |  | 45.1 | 1.94^a^ |  | 48.7 | 2.54^a^ |  | 46.0 | 2.52^a^ |
| 15 | 37.0 | 2.02, 2.34^a^ |  | 28.0 | 1.47, 2.09^a^ |  | 34.5 | 1.66, 1.92^a^ |  | 26.5 | 1.91^a^ |
| 16 | 214.2 |  |  | 22.8 | 1.39, 1.76^a^ |  | 74.3 | 4.40^a^ |  | 23.1 | 1.49, 1.85^a^ |
| 17 | 61.2 | 2.63,*d* (2.9) |  | 50.9 | 2.19^a^ |  | 56.6 | 2.41,*d*(7.5) |  | 50.8 | 2.27^a^ |
| 18 | 13.8 | 0.52, *s*, 3H |  | 11.8 | 0.54, *s*, 3H |  | 13.2 | 0.30, *s*, 3H |  | 12.1 | 0.32, *s*, 3H |
| 19 | 19.4 | 0.87, *s*, 3H |  | 19.4 | 0.85, *s*, 3H |  | 19.3 | 0.81, *s*, 3H |  | 19.3 | 0.82, *s*, 3H |
| 20 | 36.8 | 2.98,*ddd*,(12.2,8.7,3.2) |  | 47.6 | 2.76,*dd* |  | 133.9 |  |  | 134.9 |  |
|  |  |  |  |  | (10.8, 6.6) |  |  |  |  |  |  |
| 21 | 177.3 | 1.88, 2.16^a^ |  | 175.4 |  |  | 173.3 |  |  | 172.9 |  |
| 22 | 26.0 |  |  | 79.1 | 4.58,*t*(6.0*2) |  | 146.8 | 7.49, *br s* |  | 146.9 | 7.44, *br s* |
| 23 | 80.6 | 4.63,d(5.5) |  | 78.7 | 4.62,d(5.1)^,^ |  | 82.8 | 5.24, m |  | 82.7 | 5.22, m |
| 24 | 83.0 |  |  | 82.6 |  |  | 83.3 |  |  | 83.3 |  |
| 25 | 33.5 | 2.07, m |  | 30.8 | 1.91, m |  | 32.9 | 2.16, m |  | 32.8 | 2.16, m |
| 26 | 17.1 | 0.83, *d* (7.0) |  | 16.5 | 0.87, *d* (6.8) |  | 16.9 | 0.82, *d* (6.9) |  | 16.9 | 0.81, *d* (6.8) |
| 27 | 17.0 | 0.91, *d* (7.0) |  | 17.6 | 1.03, *d* (6.8) |  | 16.7 | 0.97, *d* (6.9) |  | 16.6 | 0.96, *d* (6.8) |
| 28 | 213.0 |  |  | 106.9 |  |  | 211.2 |  |  | 211.1 |  |
| 29 | 29.2 | 2.15, *s*, 3H |  | 15.0 | 1.26, *s*, 3H |  | 28.1 | 2.09, *s*, 3H |  | 28.1 | 2.09, *s*, 3H |
| Glu |  |  |  |  |  |  |  |  |  |  |  |
| 1’ | 100.9 | 4.24, *d* (8.1) |  | 100.9 | 4.23, *d* (8.1) |  | 100.9 | 4.22, *d* (8.0) |  | 100.9 | 4.22, *d* (8.1) |
| 2’ | 73.5 | 2.90^a^ |  | 73.5 | 2.90^a^ |  | 73.5 | 2.89^a^ |  | 73.5 | 2.89^a^ |
| 3’ | 76.7 | 3.10^a^ |  | 76.7 | 3.10^a^ |  | 76.8 | 3.10^a^ |  | 76.8 | 3.11^a^ |
| 4’ | 70.1 | 3.03^a^ |  | 70.1 | 3.02^a^ |  | 70.1 | 3.02^a^ |  | 70.1 | 3.02^a^ |
| 5’ | 76.8 | 3.10^a^ |  | 76.8 | 3.10^a^ |  | 76.8 | 3.10^a^ |  | 76.8 | 3.11^a^ |
| 6’ | 61.1 | 3.42, 3.65^a^ |  | 61.1 | 3.41, 3.65^a^ |  | 61.2 | 3.41, 3.65^a^ |  | 61.1 | 3.41, 3.65^a^ |
| OCH3 |  |  |  | 49.5 | 3.15, *s*, 3H |  |  |  |  |  |  |

^a^ Resonance pattern unclear due to overlapping

Table 2. Results of the cytotoxicity assay

| Sample | BT-549 |  |  | MDA-MB-231 | |  | MCF-7 |  |  | Hela |  |
| --- | --- | --- | --- | --- | --- | --- | --- | --- | --- | --- | --- |
|  | Average Absorbance | Inhibition (%) |  | Average Absorbance | Inhibition |  | Average Absorbance | Inhibition (%) |  | Average Absorbance | Inhibition (%) |
| Compound **1** | 0.2748 | 63.61 |  | 0.6102 | 28.97% |  | 0.4217 | 46.54 |  | 0.5212 | 42.05 |
| Compound **2** | 0.2836 | 62.17 |  | 0.6196 | 27.78% |  | 0.4893 | 37.07 |  | 0.6063 | 31.64 |
| Compound **3** | 0.4546 | 34.18 |  | 0.5803 | 32.74% |  | 0.4728 | 39.38 |  | 0.6464 | 26.73 |
| Compound **4** | 0.3946 | 44.00 |  | 0.5899 | 31.53% |  | 0.5301 | 31.36 |  | 0.5957 | 32.93 |
| Compound **5** | 0.4410 | 36.41 |  | 0.5734 | 33.61% |  | 0.3990 | 49.72 |  | 0.6893 | 21.48 |
| Compound **6** | 0.3510 | 51.14 |  | 0.5961 | 30.75% |  | 0.4750 | 39.08 |  | 0.5737 | 35.63 |
| Positive control | 0.1515 | 83.79 |  | 0.1789 | 83.39% |  | 0.0734 | 95.32 |  | 0.0996 | 92.70 |
| Negative control | 0.6634 |  |  | 0.8399 |  |  | 0.7540 |  |  | 0.8648 |  |
| Blank | 0.0525 |  |  | 0.0470 |  |  | 0.0400 |  |  | 0.0477 |  |

**Figures**

Fig.1. Structures of compound **1-6**

Fig.2. Major ^1^H-^1^H COSY correlations of compound **1**(bold lines)

Fig.3. Major HMBC and NOESY correlations of compound **1**

Fig.4. Major HMBC and NOESY correlations of compound **2**

Fig.5. Major HMBC and NOESY correlations of compound **3**

Fig.6. Major HMBC and NOESY correlations of compound **4**
