# Supplementary material for: Steroidal Saponins from Vernonia amygdalina Del. and Their Biological Activity
Source: Molecules. 2018 Mar 5;23(3):579. doi: 10.3390/molecules23030579 (PMC6017044; doi:10.3390/molecules23030579)
Supplement: Supplementary file 1 [file molecules-23-00579-s001.zip › molecules-269783-supple-proofread.zip!/Supporting Information Section.docx]

**Supporting Information**

**Steroidal saponins from** ***Vernonia amygdalina* Del. and their** **biological activity**

**Jing Wang^1, a^, Hua Song^1, a^, Xiaoxue Wu^a^, Shuyi Zhang^a^, Xuemin Gao^a^, Funan Li^a^, Xuan Zhu^a,^ *, Qing Chen^a,^ ***

*^a^*Department of Pharmacy, School of Pharmaceutical Science, Xiamen University, Xiamen 361102, China; diaoli187@163.com (Jing Wang); [songhua@xmu.edu.cn](mailto:songhua@xmu.edu.cn) (Hua Song); [xiaoxue_wu1@126.com](mailto:xiaoxue_wu1@126.com) (Xiaoxue Wu); [shuyi_zhang95@163.com](mailto:shuyi_zhang95@163.com) (Shuyi Zhang); [holygxm@xmu.edu.cn](mailto:holygxm@xmu.edu.cn) (Xuemin Gao); [fnlee5@xmu.edu.cn](mailto:fnlee5@xmu.edu.cn) (Funan Li).

*Correspondence: [zhuxuan@xmu.edu.cn](mailto:zhuxuan@xmu.edu.cn) (Xuan Zhu); [chenqing@xmu.edu.cn](mailto:chenqing@xmu.edu.cn) (Qing Chen)

Tel. +86-592-2881181(Xuan Zhu)

^1^ These authors contributed equally to this work and should be considered co-first authors

**Contents of Supporting Information**

| **No** | **Contents** | **Pages** |
| --- | --- | --- |
| **1** | **^1^H-NMR spectrum of vernoniamyoside A (1) in dimethyl sulfoxide-d6** | **4** |
| **2** | **^13^C-NMR spectrum of vernoniamyoside A (1) in dimethyl sulfoxide-d6** | **4** |
| **3** | **DEPT spectrum of vernoniamyoside A (1) in dimethyl sulfoxide-d6** | **5** |
| **4** | **HSQC spectrum of vernoniamyoside A (1) in dimethyl sulfoxide-d6** | **5** |
| **5** | **HMBC spectrum of vernoniamyoside A (1) in dimethyl sulfoxide-d6** | **6** |
| **6** | **^1^H-^1^H COSY spectrum of vernoniamyoside A (1) in dimethyl sulfoxide-d6** | **6** |
| **7** | **NOESY spectrum of vernoniamyoside A (1) in dimethyl sulfoxide-d6** | **7** |
| **8** | **HRESIMS spectrum of vernoniamyoside A (1) in dimethyl sulfoxide-d6** | **7** |
| **9** | **^1^H-NMR spectrum of vernoniamyoside B (2) in dimethyl sulfoxide-d6** | **8** |
| **10** | **^13^C-NMR spectrum of vernoniamyoside B (2) in dimethyl sulfoxide-d6** | **8** |
| **11** | **DEPT spectrum of vernoniamyoside B (2) in dimethyl sulfoxide-d6** | **9** |
| **12** | **HSQC spectrum of vernoniamyoside B (2) in dimethyl sulfoxide-d6** | **9** |
| **13** | **HMBC spectrum of vernoniamyoside B (2) in dimethyl sulfoxide-d6** | **10** |
| **314** | **^1^H-^1^H COSY spectrum of vernoniamyoside B (2) in dimethyl sulfoxide-d6** | **10** |
| **15** | **NOESY spectrum of vernoniamyoside B (2) in dimethyl sulfoxide-d6** | **11** |
| **16** | **HRESIMS spectrum of vernoniamyoside B (2) in dimethyl sulfoxide-d6** | **11** |
| **17** | **^1^H-NMR spectrum of vernoniamyoside C (3) in dimethyl sulfoxide-d6** | **12** |
| **18** | **^13^C-NMR spectrum of vernoniamyoside C (3) in dimethyl sulfoxide-d6** | **12** |
| **19** | **DEPT spectrum of vernoniamyoside C (3) in dimethyl sulfoxide-d6** | **13** |
| **20** | **HSQC spectrum of vernoniamyoside C (3) in dimethyl sulfoxide-d6** | **13** |
| **21** | **HMBC spectrum of vernoniamyoside C (3) in dimethyl sulfoxide-d6** | **14** |
| **22** | **^1^H-^1^H COSY spectrum of vernoniamyoside C (3) in dimethyl sulfoxide-d6** | **14** |
| **23** | **NOESY spectrum of vernoniamyoside C (3) in dimethyl sulfoxide-d6** | **15** |
| **24** | **HRESIMS spectrum of vernoniamyoside C (3) in dimethyl sulfoxide-d6** | **15** |
| **25** | **^1^H-NMR spectrum of vernoniamyoside D (4) in dimethyl sulfoxide-d6** | **16** |
| **26** | **^13^C-NMR spectrum of vernoniamyoside D (4) in dimethyl sulfoxide-d6** | **16** |
| **27** | **DEPT spectrum of vernoniamyoside D (4) in dimethyl sulfoxide-d6** | **17** |
| **28** | **HSQC spectrum of vernoniamyoside D (4) in dimethyl sulfoxide-d6** | **17** |
| **29** | **HMBC spectrum of vernoniamyoside D (4) in dimethyl sulfoxide-d6** | **18** |
| **30** | **^1^H-^1^H COSY spectrum of vernoniamyoside D (4) in dimethyl sulfoxide-d6** | **18** |
| **31** | **NOESY spectrum of vernoniamyoside D (4) in dimethyl sulfoxide-d6** | **19** |
| **32** | **HRESIMS spectrum of vernoniamyoside D (4) in dimethyl sulfoxide-d6** | **19** |

**Figure S1. ^1^H-NMR spectrum of vernoniamyoside A (1) in dimethyl sulfoxide-d6**

**Figure S2. ^13^C NMR spectrum of vernoniamyoside A (1) in dimethyl sulfoxide-d6**

**Figure S3. DEPT spectrum of vernoniamyoside A (1) in dimethyl sulfoxide-d6**

**Figure S4. HSQC spectrum of vernoniamyoside A (1) in dimethyl sulfoxide-d6**

**Figure S5. HMBC spectrum of vernoniamyoside A (1) in dimethyl sulfoxide-d6**

**Figure S6. ^1^H-^1^H COSY spectrum of vernoniamyoside A (1) in dimethyl sulfoxide-d6**

**Figure S7. NOESY spectrum of vernoniamyoside A (1) in dimethyl sulfoxide-d6**

**Figure S8. HRESIMS spectrum of vernoniamyoside A (1) in dimethyl sulfoxide-d6**

**Figure S9. ^1^H NMR spectrum of vernoniamyoside B (2) in dimethyl sulfoxide-d6**

**Figure S10. ^13^C NMR spectrum of vernoniamyoside B (2) in dimethyl sulfoxide-d6**

**Figure S11. DEPT spectrum of vernoniamyoside B (2) in dimethyl sulfoxide-d6**

**Figure S12. HSQC spectrum of vernoniamyoside B (2) in dimethyl sulfoxide-d6**

**Figure S13. HMBC spectrum of vernoniamyoside B (2) in dimethyl sulfoxide-d6**

**Figure S14. ^1^H-^1^H COSY spectrum of vernoniamyoside B (2) in dimethyl sulfoxide-d6**

**Figure S15. NOESY spectrum of vernoniamyoside B (2) in dimethyl sulfoxide-d6**

**Figure S16. HRESIMS spectrum of vernoniamyoside B (2) in dimethyl sulfoxide-d6**

**Figure S17. ^1^H NMR spectrum of vernoniamyoside C (3) in dimethyl sulfoxide-d6**

**Figure S18. ^13^C NMR spectrum of vernoniamyoside C (3) in dimethyl sulfoxide-d6**

**Figure S19. DEPT spectrum of vernoniamyoside C (3) in dimethyl sulfoxide-d6**

**Figure S20. HSQC spectrum of vernoniamyoside C (3) in dimethyl sulfoxide-d6**

**Figure S21. HMBC spectrum of vernoniamyoside C (3) in dimethyl sulfoxide-d6**

**Figure S22. ^1^H-^1^H COSY spectrum of vernoniamyoside C (3) in dimethyl sulfoxide-d6**

**Figure S23. NOESY spectrum of vernoniamyoside C (3) in dimethyl sulfoxide-d6**

**Figure S24. HRESIMS spectrum of vernoniamyoside C (3) in dimethyl sulfoxide-d6**

**Figure S25. ^1^H NMR spectrum of vernoniamyoside D (4) in dimethyl sulfoxide-d6**

**Figure S26.^13^C NMR spectrum of vernoniamyoside D (4) in dimethyl sulfoxide-d6**

**Figure S27. DEPT spectrum of vernoniamyoside D (4) in dimethyl sulfoxide-d6**

**Figure S28. HSQC spectrum of vernoniamyoside D (4) in dimethyl sulfoxide-d6**

**Figure S29. HMBC spectrum of vernoniamyoside D (4) in dimethyl sulfoxide-d6**

**Figure S30. ^1^H-^1^H COSY spectrum of vernoniamyoside D (4) in dimethyl sulfoxide-d6**

**Figure S31. NOESY spectrum of vernoniamyoside D (4) in dimethyl sulfoxide-d6**

**Figure S32. HRESIMS spectrum of vernoniamyoside D (4) in dimethyl sulfoxide-d6**
